# Does perturbed adaptive myelination contribute to cognitive dysfunction in Alzheimer’s disease?

**DOI:** 10.1371/journal.pbio.3004033

**Published:** 2026-10-12

**Authors:** Charles ffrench-Constant

**Affiliations:** Faculty of Medicine and Health Sciences, University of East Anglia, Norwich, United Kingdom; School of Medicine and Health, Technical University, Munich, GERMANY

## Abstract

The consequences of oligodendrocyte involvement in Alzheimer’s disease are poorly understood. This Perspective suggests that activity-dependent adaptive myelination, a key role of grey-matter oligodendrocytes, could be perturbed, leading to cognitive loss.

## Teaser

The consequences of oligodendrocyte involvement in Alzheimer’s disease are poorly understood. Could a key role of gray-matter oligodendrocytes—activity-dependent adaptive myelination to sustain changes in activity—be perturbed, leading to cognitive loss?

Myelination in the central nervous system (CNS)—the formation of multiple myelin sheaths around axons by oligodendrocytes—represents one of the most spectacular cell–cell interactions in biology. These sheaths hold three critical roles: enabling rapid saltatory conduction and reducing energy consumption along the axon by the localization of the voltage-dependent sodium channels to small gaps between sheaths (the nodes of Ranvier); providing metabolic support by the transfer of lactate and pyruvate from sheath to axon; and providing homeostatic support by functions such as protecting against oxidants.

Oligodendrocytes have received less attention in Alzheimer’s disease (AD) research than neurons, astrocytes or microglia. However, as highlighted in an excellent recent review [1], the evidence that oligodendrocytes have an important role in AD pathogenesis is strong. Microstructural abnormalities are seen within densely myelinated tracts in AD. Gene expression studies of the brains of patients with AD show that oligodendrocytes adopt a disease-associated state with upregulation of immune pathways and downregulation of myelination-related pathways, and genome-wide association studies searching for genes that increase risk of AD have identified a number expressed in oligodendrocytes. Oligodendrocytes generate amyloid-β (Aβ), with high levels of gene expression for amyloid precursor protein and the processing pathway components BACE1 and PSEN1. Pointing more directly to a causal role, experiments using conditional knockout strategies in mouse models of AD show that selectively preventing the ability of oligodendrocytes to generate this Aβ by removing BACE1 reduces the plaque burden by 30% (for references see [1]).

Why then have oligodendrocytes received less attention in the AD field? I would argue that this reflects, in part, a lack of attention to important differences between gray- and white-matter oligodendrocytes in the CNS. The traditional view of oligodendrocytes focuses on their role in myelinating the long projection axons in the brain and spinal cord, creating white-matter tracts. However, oligodendrocytes and their precursors are also abundant in the sparsely myelinated regions of the CNS such as the cortex; the so-called gray matter. Here, their contributions appear different to those in white matter. They generate sheaths, but detailed reconstructions in mouse cortex have shown that in many cases these only partially myelinate axons, leaving long unmyelinated stretches between sheaths [2]. Most importantly, myelin sheaths are added throughout life; in the mouse cortex, the generation of new oligodendrocytes is lifelong. The survival and integration of these new oligodendrocytes, and therefore their ability to form myelin sheaths, is dependent on activity. If activity is enhanced by sensory enrichment (e.g., hanging beads in the mouse’s environment), new oligodendrocyte integration is increased, an effect that is lost if the mouse’s whiskers are trimmed [3].

This link between survival and activity is important, as it suggests that newly formed oligodendrocytes contribute to adaptive myelination. This concept suggests that myelin sheaths can be altered in number and size by activity. This activity-dependent plasticity is hypothesized to enhance circuit function by boosting conduction and refining synchrony. In this way, myelin plasticity might contribute to long-term changes that enhance CNS function in response to activity—in other words, learning [4]. Exactly how newly formed sheaths in gray matter might enhance circuit function remains a matter of debate, given the sparse nature of myelination on many axons. However, even if it is difficult to see intuitively how sparse myelination will alter conduction speeds enough to vary synchrony, it is easier to imagine how isolated internodes could provide metabolic and homeostatic support. This provides an equally valid mechanism to enhance conduction in selected axons by enabling them to sustain activity for longer (Fig 1). The signals that regulate this adaptive process remain incompletely defined, even though the link between myelination and activity was clearly demonstrated many years ago [5].

**Fig 1:**
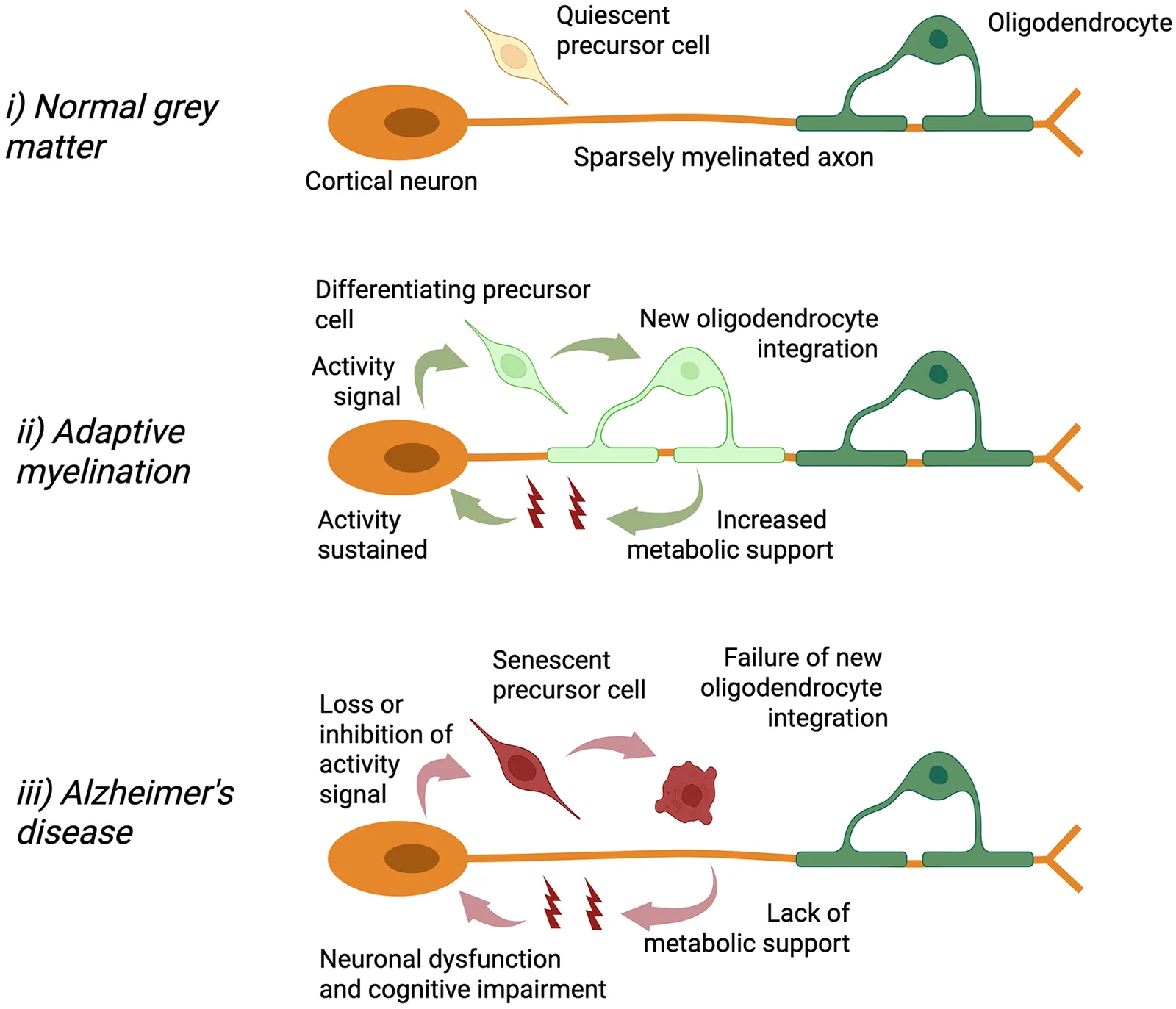
A model for the failure of adaptive myelination in Alzheimer’s disease. **(i)** Axons in normal gray matter are sparsely myelinated. **(ii)** Adaptive myelination, in which an increase in activity leads to the generation of new oligodendrocytes and/or greater integration of these cells. The increased number of sheaths supports and sustains increased activity through metabolic and/or homeostatic support (green arrows). **(iii)** In Alzheimer’s disease, multiple potential points of failure in this process (as explained in the text) may prevent new sheath formation and so set up a feedback loop (red arrows), leading to cognitive loss and neuronal dysfunction/damage due to an inability to sustain changes in activity. Figure created in BioRender.

In support of the concept of adaptive myelination, a number of experimental strategies have demonstrated a requirement for oligodendrocyte formation in experimental measures of learning in mouse models (for references see [6]). The question is therefore whether AD-associated changes might prevent the generation of new oligodendrocytes and/or myelin sheaths, and so perturb adaptive myelination? A number of lines of evidence, albeit indirect, suggest this to be the case. The downregulation of cholesterol biosynthetic pathway genes in disease-associated oligodendrocytes [7] would limit the ability of the cell to form the new sheaths essential for adaptive myelination. Aβ-plaque-associated oligodendrocyte precursors in the brains of patients with AD show a senescent phenotype that would be expected to inhibit their differentiation [8]. In the hippocampus of rodent AD models, there is a reduction in oligodendrocyte numbers without any diminution of precursor cell differentiation, here suggesting increased death of newly formed oligodendrocytes [9]. Together these findings support a model in which a failure of adaptive myelination by gray-matter oligodendrocytes due to AD-associated changes in the CNS microenvironment could contribute to the cognitive defects that characterize the disease (Fig 1). Live imaging of mouse cortex, as has been instrumental in defining the lifelong formation of gray-matter oligodendrocytes and myelin sheaths, would enable such changes to be shown directly.

This model makes the important prediction that enhancing oligodendrocyte formation may ameliorate some of the cognitive defects in AD. Here, studies attempting to promote remyelination in multiple sclerosis provide potential strategies. The anti-muscarinic drug clemastine enhances oligodendrocyte generation and remyelination, and administration shows short-term memory improvement and increased myelin in an AD mouse model [10]. Similarly, the retinoid X receptor agonist bexarotene improves cognitive loss in a mouse model [11], although this receptor has roles in other relevant cell types such as microglia that may also explain a beneficial effect. Further studies are clearly warranted here.

The model also raises a number of interesting questions. First, to what extent are oligodendrocytes primary drivers of human AD pathology? Demonstrating any causal role in snapshot studies of postmortem tissue using omics and other studies is difficult, especially given the multiple bidirectional physical and biochemical cell–cell interactions that underpin myelination. A change in one set of interactions will rapidly result in changes in others, establishing a feedback loop leading to further perturbation of oligodendrocyte formation and function (Fig 1). This also predicts that enhancing oligodendrocyte function may improve cognitive performance in AD, even if oligodendrocyte pathology is not the causal event.

Second, does it explain the intriguing observation that the regions of the CNS that are myelinated at a later stage of human development show the first evidence of neurofibrillary tangles [12]? Myelination in these regions may result from adaptive myelination to a much greater extent than regions that are earlier and more densely myelinated, where intrinsic neurodevelopmental mechanisms likely make a greater contribution. It follows that these later myelinating regions will be more sensitive to pathologies resulting from the loss of metabolic and homeostatic support once adaptive myelination fails.

Third, do individual differences in oligodendrocyte pathology contribute directly to AD heterogeneity? Based on studies in tissue from patients with multiple sclerosis, this seems likely. Here, RNA sequencing studies show distinct patterns of oligodendrocyte cellular pathology, with all cells across different lesions in a single individual expressing the same pattern whatever their location or stage of pathological evolution [13]. Given the potential effect of AD risk genes on myelination (e.g., *APOE4* through impaired lipid delivery to oligodendrocytes [14]), one would predict that distinct pathological pathways will emerge within oligodendrocytes in AD, with the contribution of each varying from patient to patient depending on their genotype and on confounding pathologies such as neuroinflammation and vascular disease. From this it follows that biomarker studies to identify potential pathways in each patient will be required to prevent a ‘one size fits all’ approach to oligodendrocyte-targeted treatments. The danger otherwise would be that new therapies being assessed in trials might be discarded, not because they failed to work but because their effect was masked by lack of efficacy in patients with quite distinct cellular pathologies.

Whatever the answers to these questions, their study will generate important new knowledge about gray-matter oligodendrocytes—a cell type the unique biology of which deserves greater attention from the AD research community.

## Abbreviations

AD: Alzheimer’s disease
Aβ: amyloid-β
CNS: central nervous system
